# The progress of microenvironment-targeted therapies in brain metastases

**DOI:** 10.3389/fmolb.2023.1141994

**Published:** 2023-03-28

**Authors:** Lifu Long, Zhenjie Yi, Yu Zeng, Zhixiong Liu

**Affiliations:** ^1^ Department of Neurosurgery, Xiangya Hospital, Central South University, Changsha, HN, China; ^2^ XiangYa School of Medicine, Central South University, Changsha, HN, China; ^3^ National Clinical Research Center for Geriatric Disorders, Xiangya Hospital, Central South University, Changsha, HN, China

**Keywords:** brain metastases, brain microenvironment, targeted therapy, immunotherapy, glioma, microenvironment-targeted therapy

## Abstract

The incidence of brain metastases (BrM) has become a growing concern recently. It is a common and often fatal manifestation in the brain during the end-stage of many extracranial primary tumors. Increasing BrM diagnoses can be attributed to improvements in primary tumor treatments, which have extended patients’ lifetime, and allowed for earlier and more efficient detection of brain lesions. Currently, therapies for BrM encompass systemic chemotherapy, targeted therapy, and immunotherapy. Systemic chemotherapy regimens are controversial due to their associated side effects and limited efficacy. Targeted and immunotherapies have garnered significant attention in the medical field: they target specific molecular sites and modulate specific cellular components. However, multiple difficulties such as drug resistance and low permeability of the blood-brain barrier (BBB) remain significant challenges. Thus, there is an urgent need for novel therapies. Brain microenvironments consist of cellular components including immune cells, neurons, endothelial cells as well as molecular components like metal ions, nutrient molecules. Recent research indicates that malignant tumor cells can manipulate the brain microenvironment to change the anti-tumoral to a pro-tumoral microenvironment, both before, during, and after BrM. This review compares the characteristics of the brain microenvironment in BrM with those in other sites or primary tumors. Furthermore, it evaluates the preclinical and clinical studies of microenvironment-targeted therapies for BrM. These therapies, due to their diversity, are expected to overcome drug resistance or low permeability of the BBB with low side effects and high specificity. This will ultimately lead to improved outcomes for patients with secondary brain tumors.

## 1 Introduction

The prevalence of brain metastasis (BrM) is approximately 8.5%–9.6%. However, this likely underestimates the true incidence of BrM because many cancer patients with this condition are not included within the statistics. BrM is becoming more frequent even more frequent than any other primary tumor in the brain, with lung cancer having the highest incidence at (19.9%), followed by melanoma (6.9%), renal cancer (6.5%), breast cancer (5.1%), and colorectal cancer (1.8%) (Valiente et al., 2018). The increased incidence of BrM may be attributed to the prolonged overall survival (OS) of patients with cancer because of novel cancer therapies. Current medical treatments for BrM primarily include systemic chemotherapy, targeted therapy, and immunotherapy (Achrol et al., 2019). Traditional systemic chemotherapeutic agents such as pemetrexed and temozolomide have a limited role in management of BrM due to the BBB or the blood-tumor barrier (BTB). Targeted therapy has dramatically improved the OS of patients with BrM (Valiente et al., 2018), for instance, osimertinib targeting epidermal growth factor receptor (*EGFR*) mutated non-small cell lung cancer (NSCLC) BrM, lapatinib targeting human epidermal growth factor receptor 2 (HER-2) mutated breast cancer, and B-Raf kinase gene (BRAF) inhibitor dabrafenib combined with the mitogen-activated protein kinase (MAPK) pathway inhibitor trametinib targeting melanoma (Achrol et al., 2019). Additionally, novel drugs targeting immune checkpoints, including cytotoxic T-lymphocyte-associated protein 4 (CTLA-4) (ipilimumab), programmed cell death protein 1 (PD-1) (pembrolizumab and nivolumab), and programmed death-ligand 1 (PD-L1) (atezolizumab), also have incredibly prolonged OS of BrM patients (Achrol et al., 2019).

Despite advancements in BrM therapy, there are significant challenges such as drug resistance and low permeability of the BBB. These challenges have led to growing concerns in the development of novel therapies with high efficacy. Cancer metastasis is a complex process involving multiple interactions and mechanisms, including but not limited to dissemination, intravasation, circulating, extravasation, and colonization (Fares et al., 2020). The unique microenvironment of BrM promotes the expansion of tumor cells and protects them from anti-tumoral insults. This specific microenvironment mainly includes cellular and non-cellular components, the former being the focus of recent research. Cellular components include astrocytes, neurons, tumor-infiltrating lymphocytes, tumor-associated macrophages, natural killer cells (Tomaszewski et al., 2019). Non-cellular components mainly refer to the extracellular matrix (ECM) and BBB (Achrol et al., 2019). The ECM includes nutrients such as oxygen and electrolytes such as iron and calcium ions, exosomes, etc., (Hoshino et al., 2015; Basnet et al., 2019; Chi et al., 2020). Despite the emergence of many cutting-edge therapeutic targets and drugs for various types of BrM, the therapeutic effects have been unsatisfactory. This can be attributed to the complex microenvironment of BrM, which poses significant challenges for effective treatment. Firstly, the BBB separates the brain from the peripheral circulation in the microenvironment, preventing the drugs that target the primary tumor from penetrating the brain tissue. Secondly, the structure of the BBB changes after brain metastasis, forming the blood-tumor barrier (BTB). Then the BTB makes it difficult for drugs that initially passed through the BBB to enter the brain tissue (Achrol et al., 2019). Thirdly, BrM is heterogeneous, with differences in the molecular traits of primary tumors and metastases leading to a loss of original targets of cancer cells (Achrol et al., 2019). Furthermore, the microenvironment of metastases is also heterogeneous with its microenvironment modified by metastatic cells which differ from the primary tumour (Mansfield et al., 2016; Priego et al., 2018; Kim et al., 2019; Kudo et al., 2019; Vareslija et al., 2019; Sirkisoon et al., 2020). This renders drugs that were initially effective against the primary tumor microenvironment ineffective. Additionally, it was previously believed that the brain was ‘immune privileged. However, recent research has shown that immune cells exist in the brain microenvironment (Jiang et al., 2020), and their role in promoting metastatic tumor outgrowth is still being studied in detail (Jiang et al., 2020).

The microenvironmental therapy that we are proposing includes a series of therapies which target components of the BrM microenvironment or inhibit the interaction between cancer cells and the microenvironment components to inhibit tumor growth. This is a different strategy to traditional chemotherapy in that it acts precisely on specific cells or molecules as well as not directly targeting and killing cancer cells and through induced cytotoxicity. Although this therapy is still being debated regarding its efficacy and side effects, it has been demonstrated in cell experiments to have advantages in overcoming drug resistance or low permeability of the BBB with low side effects and high specificity (Tomaszewski et al., 2019). This therapy showed promising drug efficacy when administered alone and can also be combined with other traditional drugs for improved effectiveness (Tomaszewski et al., 2019).

In addition, single cell sequencing technology develop fast. In the field of BrM, this technology gives a deep insight into the expression of specific cells in BrM microenvironment (Gonzalez et al., 2022). Single cell sequence not only analyses the genomic and proteomic profile of BrM microenvironment, especially immune cells, but also provides various prognosis and treatment approaches (Parker et al., 2020; Gonzalez et al., 2022; Jin et al., 2022). It is believed that development of single cell sequencing technology will contribute to the exploration in BrM microenvironment (Gonzalez et al., 2022).

It is important to note that microenvironment therapy must consider the heterogeneity of BrM. Previous metastatic cancer antagonists were designed based on the assumption that primary tumor and metastases are the same on a molecular level; however, they differ significantly (Valiente et al., 2018). Microenvironment targeted therapy has shown promising prospects in the preclinical and clinical stages, but there are still substantial unexplored fields with many medicine designs still in therapeutic setting. In this review, we highlight the uniqueness of the BrM microenvironment and summarize the research progress of microenvironment targeted therapy according to cell types, including astrocytes, macrophages and microglia, T lymphocytes, and endothelial cells, and other components.

## 2 The uniqueness of BrM microenvironment

The tumor microenvironment is essential for tumor cells to survive. It undergoes dynamic cellular and non-cellular components changes, which could promote the heterogeneity of cancer cells, clonal evolution, and drug resistance. The microenvironment of BrM differs from that of the primary brain tumors. Understanding these differences between BrM may aid in the development of targeted treatments strategies for different types of BrM as well as drugs with broad anti-cancer capabilities for common targets.

### 2.1 Differences in brain microenvironment among BrMs coming from different primary sites

BrMs from different primary sites experience differences at both the molecular and cellular level. For example, BrMs from lung cancer, breast cancer, and colon cancer. BrM in lung cancer showed significantly higher expression of commonly mutated genes, especially the genes regulating checkpoint pathways [tumor necrosis factor receptor superfamily 9 (TNFRSF9), tumor necrosis factor receptor superfamily 4 (TNFRSF4), programmed cell death 1 ligand 2 (PDCDILG2), indoleamine 2,3-dioxygenase 1 (IDO1), inducible T Cell costimulator (ICOS), cluster of differentiation 274 (CD274)] and lymphocyte infiltration [T cell receptor gamma alternate reading frame protein (TARP), protein tyrosine phosphatase receptor type c (PTPRC), protein tyrosine phosphatase non-receptor type 7 (PTPN7), interleukin 10 receptor subunit alpha (IL10RA), granzyme l (GZMK), cluster of differentiation 52 (CD52), cluster of differentiation (CD2), C-C motif chemokine receptor 5 (CCR5), C-C motif chemokine ligand 5 (CCL5)], indicating that lung cancer brain metastasis may be more sensitive to immunotherapy (Jiang et al., 2020). In contrast, BrM from melanoma have higher presence of CD4 and CD8 positive T cells, while BrM in breast cancer has a higher presence of neutrophils and macrophages, indicating that breast cancer BrM may show increased drug resistance compared with melanoma BrM (Klemm et al., 2020; Gonzalez et al., 2022). Besides, lung cancer BrM has more T lymphocytes, while melanoma and oval cancer have more B lymphocytes (Gonzalez et al., 2022). Through single cell sequencing technology, Sudmeier, et al. also found that CD8^+^ T cell phenotype is linked with spatial distribution within the tumor (Gonzalez et al., 2022). Additionally, hypoxia was found to be a characteristic in lung cancer BrM compared with other types of BrMs (Corroyer-Dulmont et al., 2021). Despite differences in the microenvironment of BrMs from different primary sites, several similarities have been found. *In vivo* experiments have reported that both breast cancer cells and lung cancer cells interact with astrocytes in the central nervous system to improve the expression of survival genes, including B-cell lymphoma-2-like protein 1 (BCL2L1), twist family BHLH transcription factor 1 (TWIST1), and glutathione S-transferase alpha 5 (GSTA5) to obtain drug resistance. (Table 1) (Kim et al., 2011; Vareslija et al., 2019). These differences in the microenvironment of BrMs from primary sites have important implications for treatment options and highlight the need for tailored therapies.

**TABLE 1:** Comparison between BrMs from different primary sites. Checkpoint pathways genes include genes of TNFRSF9, TNFRSF4, PDCDILG2, IDO1, ICOS, CD274. Lymphocyte infiltration genes include genes of TARP, PTPRC, PTPN7, IL10RA, GZMK, CD52, CD2, CCR5, CCL5. “-” represents that the value is relatively normal compared with the decreased or increased one. “/” means no outcomes available. “↑” means overexpression. “↓” means relatively low expression.

BrMs from different primary sites				
	NSCLC BrM	Breast cancer BrM	Melanoma BrM	Colon cancer BrM
Checkpoint pathways genes	↑	-	-	-
Lymphocyte infiltration genes	↑	-	-	-
CD4^+^ and CD8+T cells	—	-	↑	-
Neutrophils	—	↑	-	-

### 2.2 Differences between BrMs and glioma

Primary and metastatic tumors, such as gliomas and BrM have distinct differences in tumor microenvironment. For example, Klemm, et al. reported that gliomas contain many tumor-associated macrophages (TAMs), but have few T cells, especially in isocitrate dehydrogenase (IDH)-mutated tumors. However, lymphocytes and neutrophils are abundant in BrM, and the microenvironment of BrM contains more immune regulatory factors including CD40L, interleukin 6 receptor (IL6R), inhibin subunit beta a (INHBA), and amphiregulin (AREG) according to experiment data from Klemm, et al. (Klemm et al., 2020). Plasma tumors (especially IDH mutant gliomas) have a lower presence of immune cells (Klemm et al., 2020) (Table 2). However, data from Sun, et al. showed different results. The experiment showed that lung cancer BrM contains more macrophages, T lymphocytes, and mastocytes, while gliomas contains more astrocytes and microglia (Perelroizen et al., 2022; Sun et al., 2022), possibly because microenvironment can change as the glioma progression goes on (increases in macrophages, lymphocytes, monocytes and NK cells over time) (Varn et al., 2022; Yeo et al., 2022). Besides, subclones of stroma cells in glioma were found to be much more than that in BrM (Schaettler et al., 2022). This is possibly because gliomas arise from the slow accumulation of somatic mutations, while BrM likely develop quickly from the rapid growth of an already transformed subclone upon arrival into the central nervous system (CNS). This malignant clone will quickly develop into an apparent tumor, allowing less time for the development of genetically disparate subclones (Schaettler et al., 2022).

**TABLE 2:** Comparison between BrMs and glioma. NSCLC represents non-small cell lung cancer.

	BrMs					Intracranial primary cancer
	NSCLC BrM	Breast cancer BrM	Melanoma cancer BrM	Colon cancer BrM	Liver cancer BrM	Glioma
CD4^+^ and CD8+T cells	—	-	↑	-	—	↓
Neutrophils	—	↑			—	-
TAMs	↑	-			—	↑

### 2.3 Differences in microenvironments between primary tumor sites and the matched BrMs

Differences in immune microenvironments between primary tumor and BrM, especiallyNSCLC, have been explored. In NSCLC BrM, dendritic cell maturation procession and leukocyte extravasation signalling pathways in innate immunity are inhibited, while tumor-associated macrophages are increased. Regarding specific immunity, PD-1+ lymphocytes, helper T cells 1, and PD-L1+ immune cells are reduced in BrM, which may explain why PD-L1 targeted immunotherapy is a poor therapeutic strategy for patients with BrM. Interestingly, due to the accordance of antigen between BrM and primary tumor, T cell receptor (TCR) repertoire remains relatively unchanged (Mansfield et al., 2016; Kim et al., 2019; Kudo et al., 2019). Additionally, astrocytes which express signal transducer and activator of transcription 3 (STAT3) contribute to BrM by regulating innate and specific immunity as well as establish gap junctions with metastases cells to enhance the growth ability and drug resistance of tumor cells (Chen et al., 2016; Sato et al., 2017; Priego et al., 2018; Kim et al., 2019; Song S. G. et al., 2021; Song Z. et al., 2021). When compared with primary breast cancer, breast cancer BrM tends to have lower infiltration of immune cells (macrophages, microglia, lymphocytes, and monocytes), lower protein and gene expression of immune activation markers (CD27, T cell immunoglobulin and mucin-domain containing-3 (Tim-3), and CD137), and lower expression of immune-related genes (PD-L1 and CTLA-4) (Schlam et al., 2021; Giannoudis et al., 2022). In melanoma BrM, oxidative phosphorylation (OXPHOS) gene set in Kyoto Encyclopedia of Genes and Genomes (KEGG) database is enriched compared with primary cancer (Fischer et al., 2019), while melanoma BrM has lower T-cell content and microvessel density (Weiss et al., 2021). Besides, Sato, et al. found several genes upregulation in breast cancer and melanoma BrM compared with their primary tumors. In breast cancer BrM, cell cycle dysregulation genes [E2F transcription factor 3 (E2F3) and retinoblastoma protein (RB)], proto-oncogenes [Kirsten rat sarcoma virus (KRAS) and anaplastic lymphoma kinase (ALK)], kinase-driven pathways genes [src proto-oncogene (SRC), mechanistic target of rapamycin kinase (mTOR) and HER2], metastases formation-associated genes [C-X-C motif chemokine receptor 4 (CXCR4), plasmolipin (PLLP), tumor necrosis factor superfamily member 4 (TNFSF4), vascular cell adhesion molecule 1 (VCAM-1), solute carrier family 8 member a2 (SLC8A2), and solute carrier family 7 member a11 (SLC7A11)] were overexpressed (Chen et al., 2016; Song S. G. et al., 2021; Song Z. et al., 2021). Only metastasis formation-associated genes were upregulated in melanoma BrM compared with melanoma primary tumor (Sato et al., 2017). Nevertheless, immunohistochemical similarity of immune cells including tumor infiltrating lymphocytes (TIL) and TAMs is reported in gynaecological malignancies/renal cell carcinoma and their matched BrM (Gill et al., 2021; Steindl et al., 2021) (Table 3).

**TABLE 3:** Comparison between BrMs and primary tumors. Anti-inflammatory markers include TOLLIP, HLA-G. Cell cycle dysregulation genes include E2F3 and RB. Proto-oncogenes stand for KRAS and ALK. Kinase-driven pathways represent SRC, mTOR and HER2. Metastasis formation-associated genes include CXCR4, PLLP, TNFSF4, VCAM1, SLC8A2, and SLC7A11. BrM/primary tumor represents relative relationship between BrM and primary tumor. TAMs, tumor-associated macrophages. NSCLC, non-small cell lung cancer.

	NSCLC BrM/primary tumor	Breast cancer BrM/primary tumor	Melanoma BrM/primary tumor
PD-1+ and PD-L1+ T cells	↓	—	—
TAMs	↑	—	—
Dendritic cell maturation	↓	—	—
Leukocyte extravasation	↓	—	—
TCR clonality	↑	—	—
Anti-inflammatory markers	↑	—	—
Interferon-γ-related gene signature	↓	—	—
Cell cycle dysregulation genes	—	↑	—
Proto-oncogenes	—	↑	—
Kinase-driven pathways genes	—	↑	—
Metastasis formation-associated genes	—	↑	↑

### 2.4 Differences in microenvironments between intracranial metastases and extracranial metastases

There are many differences in microenvironment between intracranial metastases and extracranial metastases, including differences in physical condition, immune cells, extracellular matrix, and exosomes.

#### 2.4.1 Physical condition

Physical conditions also play a role in molecular expression in metastasis with different organotropism. For example, breast cancer lung metastasis has an increased expression of mitochondrial electron transport Complex I, oxidative stress, and counteracting antioxidant programs compared to primary breast cancer and breast cancer brain metastasis, which may be related to more oxidative stress in the lung (Basnet et al., 2019; Hebert et al., 2020; Weiss et al., 2021).

#### 2.4.2 Immune cells

Immune cells in BrM, such as CD4 and CD8 positive T cells, are fewer compared to extracranial metastases. However, other immune cells such as B cells and macrophages do not show significant differences. Fewer T lymphocytes lead to less immunoediting of metastatic cells, which may contribute to the lower expression of tyrosinase in BrM (Bartlett et al., 2014). Although there are fewer T lymphocytes in BrM, intracranial and extracranial metastases have similar responses to immune checkpoint inhibitors, indicating that T lymphocytes may not play a crucial role in metastasis. Additionally, BrM cells have been found to have an unstable gene expression and a neural-like cell state, which suggests that BrM cells adapt to the BrM microenvironment through interaction with intracranial stromal cells (Bartlett et al., 2014).

#### 2.4.3 Extracellular matrix

The protein in ECM is composed of both tumor and stromal cell-derived proteins. This is due to the influence of tumor cells on stromal cell protein synthesis. This is due to the influence of tumor cells on stromal cell protein synthesis. Common protein expressions in the ECM of BrM include s100 calcium binding protein a4 (S100A4), annexin a2 (ANXA2), transforming growth factor beta 1 (TGFB1) and CCR2, while different protein expressions include more serpin family B member 1 (SERPINB1) in the brain (Hebert et al., 2020; Weiss et al., 2021).

#### 2.4.4 Exosome

Articles reported that exosomes expressed by tumor cells could guide tumor metastasis to organotropism (Hoshino et al., 2015). Different integrins (ITGs) on exosomes determine the organotropism of tumor metastases. The type of integrins is different in exosomes with different organotropism and specific cells living in the targeted organs will absorb these exosomes for more precise organ targeting. For example, ITGα6β4 and ITGα6β1 are associated with lung metastasis, while ITGαvβ5 is associated with liver metastasis. ITGβ3 was separated from BrM cells *in vitro*, and exosomes from 831-BrT cells were absorbed by cerebral epithelial cells specifically (Hoshino et al., 2015) (Table 4).

**TABLE 4:** Comparison between BrMs and extracranial metastasis. ITG, integrin.

Breast cancer					Melanoma	
	Brain metastasis	Liver metastasis	Lung metastasis	Bone metastasis	Brain metastasis	Extracranial metastasis
Characterized tumor cell-derived proteins	CD109, SERPINB1, HCFC1 and cerebellin-1	COL6A5	collagen COL4A4 and laminin-121	S100A6 and S100A11	—	—
Characterized stroma cell-derived proteins	Secreted neuronal glycoprotein Lgi1, Adam22 and brevican	Tnc, Fn1, fibrinogens, thrombin and von Willebrand factor	Laminin chains, type IV collagens and pulmonary surfactantassociated protein A1	Thrombospondin-1, another S100 protein, the protease cathespin-G, the protease inhibitors cystatin C and stefin-2	—	—
Exosome integrins	ITGβ3	ITGαvβ5	ITGα6β4 and ITGα6β1	—	—	—
CD4^+^ and CD8^+^ T cells	↓	-	-	-	↓	-
Overall extracellular matrix protein quantity	↓	-	-	-	—	—
Glycoproteins proportion	-	↑	-	-	—	—
Immunosuppression	—	—	—	—	↑	—
Oxidative phosphorylation	—	—	—	—	—	↑

## 3 Therapies targeting BrM microenvironment

Previous research focuses primarily on genomic, epigenomic, and transcriptomic landscapes of cancer cells (Achrol et al., 2019). Understanding the microenvironments and protein expression of different types of cancer can lead to significant improvements in cancer therapies, such as targeted therapies. This has the potential to significantly improve survival rates of cancer such as driver gene mutated lung adenocarcinoma, breast cancer, and melanoma. However, most cancers still develop resistance to this treatment strategy, highlighting the importance of further research in molecular communication between cancer cells and the surrounding microenvironment. However, relatively limited studies focus on the treatment by targeting the microenvironment of metastatic brain tumors. Targeting the cellular components of the microenvironment of BrM can be used as a novel strategy to treat cancer.

### 3.1 Targeting astrocytes

The interaction between astrocytes and cancer cells can occur directly through the gap junction, paracrine, exosome, and certain gene regulations like STAT3. These signalling pathways can be useful drug targets.

#### 3.1.1 Targeting gap junction

One example is targeting gap junctions*:* astrocytes can establish gap junctions with metastatic cancer cells and activate downstream signalling pathways to promote metastasis formation. Protocadherin 7 (PCDH7) on the cell membranes of these 2 cells interact with each other first to act as an “anchor,” and then the connexin 43 (Cx43) molecules on the two membranes are connected to form a gap junction for molecular exchange (Chen et al., 2016). This can then promote cancer metastasis through three pathways: the cyclic guanosine monophosphate–adenosine monophosphate (cGAMP) pathway, calcium ion pathway, and endothelin pathway.

The cGAMP pathway refers to the transfer of cGAMP from cancer cells to astrocytes through gap junctions, stimulating astrocytes to express interferon (IFN) and tumor necrosis factor alpha (TNF-α). These act directly on the surface receptors of cancer cells through the paracrine system, and the downstream pathways such as STAT1 and nuclear factor kappa b (NF-κB) pathways enhance the proliferation and drug resistance of cancer cells. Meclofenamate and tonabersat are Cx43 gap junction blockers, effectively inhibiting *in vitro* cancer metastasis (Chen et al., 2016).

Calcium ions play a complex role in modulating tumor cell apoptosis. Research has found that persistent increases in calcium benefit tumor cell survival, while transient increases lead to apoptosis. The calcium ion pathway refers to the persistent transmigration of calcium ions from astrocytes into cancer cells through gap junctions which leads to an increase in the activity of phosphodiesterase beta (PLCβ) in the cancer cell cytoplasm. PLCβ hydrolyzes phosphatidylinositol 4,5-bisphosphate (PIP2) into inositol 1,4,5-trisphosphate (IP3) and 1,2-diacylglycerol (DAG). These molecules store the transiently increased calcium ions in the cell to form a continuously high calcium intracellular environment. These calcium ions promote the activation of calcium-dependent molecules, such as calmodulin-dependent protein kinase II (CaMK2) and S100A4. These promote cancer metastasis. Studies involving the inhibition of PLC with drugs like edelfosine inhibit cancer metastasis in mouse models (Biermann et al., 2022).

Nevertheless, the report implicated that calcium chelator effectively limited BrM by restraining ionic calcium concentrations in the cytoplasm of *in vitro* tumor cells. One theory is that this resulted in a transient concentration increase of ionic calcium and subsequent tumor cell apoptosis (Fischer et al., 2019). The various results of increased ionic calcium concentration on cancer cells are not fully understood. It is likely that different signalling pathways or astrocyte and cancer cell interactions may be involved. Further research is needed to explore this mechanism and understand the underlying causes. In the endothelin pathway, the expression of interleukin-6 (IL-6) and interleukin-8 (IL-8) molecules in cancer cells increases when gap junctions are established. These molecules promote the expression of endothelin and its receptors [endothelin receptor a (ETAR) and endothelin receptor b (ETBR)] on astrocytes through paracrine signalling. Cancer cells stimulated by endothelin activate the AKT/MAPK pathway, which enhances their drug resistance (Kim et al., 2014). A dual endothelin receptor antagonist called macitentan has been found to increase the effectiveness of the drug paclitaxel in treating BrMs from breast and lung cancer in mice model (Lee et al., 2016). However, clinical trials have not been seen. Further studies are needed.

#### 3.1.2 Targeting paracrine

Astrocytes can also reciprocally transmit signals to cancer cells through direct small molecule signalling. Cancer cells secrete molecules such as macrophage migration inhibitory factor (MIF), interleukin-8 (IL-8), and plasminogen activator inhibitor-1 (PAI-1) subsequently activating astrocytes. Astrocytes then secrete IL-6, TNF-α, IL-1β, IL-23, and other signalling molecules that promote cancer cell proliferation (Jaffe, 2005; Lee et al., 2016; Ren et al., 2018). IL-3 plays a vital role in the extravasation of cancer cells by promoting the production of matrix metallopeptidase 2 (MMP-2) by cancer cells, enhancing the invasiveness of cancer cells (Fischer et al., 2019). Paracrine signalling between these 2 cells is not necessarily reciprocal, it can be unilateral instead. Another article found that estradiol activates astrocytes during triple-negative breast cancer (TNBC) metastasis, activating astrocytes, promoting metastasis. This pathway promotes the expression of brain derived neurotrophic factor (BDNF) and epidermal growth factor (EGF) after estradiol binds to the intracellular receptors of astrocytes. BDNF and EGF act on downstream receptors [tropomyosin receptor kinase b (TrkB) and EGFR] on the surface of cancer cells and activate pathways ensuring cancer cell invasiveness (Embi et al., 2012; Kim et al., 2014). Anti-IL-6 receptor antibody tocilizumab (Lee et al., 2016), IL-23 neutralizing antibody, and anti-TrkB antibody ANA-12 have been identified as possible drugs in the treatment of metastatic cancer in mice model (Lee et al., 2016). Further toxicity tests and clinical trials may come into sight in the future.

#### 3.1.3 Targeting exosome

The exosome pathway is a recently discovered mechanism by which astrocytes can promote cancer growth. Astrocytes deliver PTEN-targeted miRNAs to metastatic cancer cells through exosomes, reducing PTEN expression. This leads to increased expression of chemokine CCL2 in cancer cells and recruits many myeloid cells (microglia and macrophages) that are ionized calcium binding adaptor molecule 1 (IBA1) positive. The latter cells can promote cancer cell proliferation and reduce apoptosis. Targeting PTEN, exosomes, and CCL2 through drug development may be a potential therapeutic strategy for treating BrM(Seike et al., 2011; Klein et al., 2015).

#### 3.1.4 Targeting STAT3

Astrocytes can also promote cancer growth by affecting the immunity in the brain microenvironment. Neibla Priego et al. found that STAT3-positive astrocytes can inhibit the anti-tumor effect of CD8-positive T cells and promote aggregation of CD74-positive cells (macrophages and microglia) to interfere with innate immunity. Silibinin (trade name Legasil) is a drug that specifically binds to the src homology 2 (SH2) domain on the pSTAT3 molecule of astrocytes, inhibiting the action of STAT3, and enhancing the efficacy of chemotherapy drugs in brain tumors when used in combination (Priego et al., 2018; Contreras-Zarate et al., 2019). Case report of Silibinin used in BrM was reported by Neibla, et al. (Priego et al., 2018). As silibinin has already been applied in clinical trials in other diseases like hepatitis and breast cancer, the prospect of silibinin can be promising (Ferenci et al., 2008; Lazzeroni et al., 2016).

### 3.2 Targeting macrophages and microglia

Unlike astrocytes, macrophages promote tumor BrM by affecting the ability or number of other immune cells. Therefore, drug treatments should aim to reduce the number of macrophages in the brain or to change the expression of macrophages in the brain.

#### 3.2.1 PI3K

Phosphoinositide 3-kinase (PI3K) promotes BrM by driving the differentiation of macrophages into a tumor-promoting phenotype. PI3K is a master regulator of brain-metastases-promoting macrophages and microglia. The infiltration of marrow-derived-macrophages (MDM) is inhibited by PI3K inhibitor buparlisib, and the MDM-induced tumor invasion can be reduced significantly (Caetano et al., 2016). PI3K inhibitor buparlisib can reduce both macrophage/microglia induced metastases, implicating those microglia involved in metastases promotion (Benbenishty et al., 2019).

#### 3.2.2 CSF-1

Colony stimulator factor-1 (CSF-1) plays a crucial role as a growth factor of macrophages and microglia in a paracrine loop between metastases cells and monocyte-derived cells. Although studies have shown that anti-CSF-1 (5A1, a CSF-1 antagonist) treatment effectively limits macrophage proliferation in BrM *in vitro*, an alternative CSF-1 ligand (IL-34) released by both metastatic cells and normal brain microenvironment can have a CSF-1-like effect rendering anti-CSF-1 treatment ineffective. Therefore, when considering treatment options, targeting IL-34 in addition to anti-CSF-1 treatment should be taken into account in order to achieve optimal results (Noda et al., 2012).

#### 3.2.3 LncRNA

Long non-coding RNAs (lncRNAs) have emerged as a new target in BrM research. LncRNAs are expressed by metastatic cells and activate a signalling pathway involving Janus kinase 2 (JAK2), oncostatin M, IL-6, and STAT3 to increase the expression of ICAM-1 and CCL2. This enhances the ability of the cancer cells to attach to capillary endothelial cells and recruit macrophages. These recruited macrophages can further promote lncRNA expression by releasing oncostatin M and IL-6. Depletion of Lnc-BM with nanoparticle-encapsulated siRNAs in mice model effectively treated BrM^51.^ Therefore, developing therapies that target oncostatin M and IL-6 or directly target lncRNA may be a promising strategy for treating BrM (Zhang et al., 2015).

#### 3.2.4 Toll-like receptor 9

Microglia are a type of immune cell found in the brain microenvironment which exert anti-metastases effects. They are activated by CpG oligodeoxynucleotides (CpG-ODN), a toll-like receptor (TLR) 9 agonist, causing the microglia to display an increased expression of anti-tumor genes, inducing apoptosis of metastases cells by direct contact with tumor or early metastases focus (Rietkotter et al., 2015). Although inhibition of primary tumor was proved to be valid in CpG ODN administration, Xiong, et al. found that in mice model, the therapeutic effect was limited in treating BrM, due to the increase in regulatory T lymphocytes (T_reg_s) in BrM (Marabelle et al., 2013). T_reg_s play an important role in immune suppression in BrM. The suppression of other immune cells like microglia may be the reason why CpG ODN cannot limit the BrM progression (Xiong et al., 2008; Marabelle et al., 2013).

#### 3.2.5 Nanocarrier

Drug delivery *via* nanocarrier is a new direction in current research for BrM treatment. Nanocarrier-mediated drug delivery is a promising approach that could lead to the development of more effective and targeted therapies for BrM in the future. This new technology has several advantages over traditional drugs: multiple signal molecules can be set on the membrane of nanoparticles allowing for multiple cells to be targeted, the nanoparticle can carry a great amount of traditional anti-tumor drugs once completed decreasing time spent on finding drug targets and nanoparticles (unlike brain tumor antagonists) have a more complicated design allowing them to cross the BBB more effectively (Zhang et al., 2019).

Tian Zhang et al. designed a nanoparticle, iRGD-terpolymer-lipid hybrid nanoparticle with coloaded doxorubicin and mitomycin c (iRGD-DMTPLN), with an iRGD signal molecule iRGD on it, which targets endothelial and BrM cells as well as TAMs. After interacting with endothelial cells, the particle can be endocytosed by the cells, transferred from peripheral circulation to the brain microenvironment, and then be endocytosed by TAMs and tumor cells, releasing the contained drug. This nanoparticle effectively reduces metastases burden and TAMs, significantly restoring the brain microenvironment (Zhang et al., 2019).

Pengfei Zhao et al. designed a T12 peptide-modified albumin nanoparticle coloaded with regorafenib and disulfiram/copper ion chelant. This particle also has multiple target cells, including brain tumor capillary endothelial cells, tumor vessel endothelial cells, cancer cells, and TAMs. The drug acting on TAMs repolarized M2Φ into M1Φ instead of killing macrophages (Zhao et al., 2021).

#### 3.2.6 Ionic iron

Macrophages have both an indirect and direct pathway to affect tumor outgrowth*.* In Leptomeningeal metastases (LM), inflammatory cytokines secreted from macrophages induce iron-binding protein lipocalin-2 (LCN2) and its receptor solute carrier family 22 member 17 (SCL22A17) expression in metastatic cells. Tumor cells can then obtain ionic iron from the extracellular matrix and contribute to chemical synthesis. An experiment in a mouse model showed that iron chelator deferoxamine inhibited LM (Chi et al., 2020). Microglia also have a role in metastasis formation, so their inhibition is crucial in the treatment of BrM.

### 3.3 Targeting T lymphocytes

T lymphocytes are critical in the anti-tumor effect but are constantly inhibited by tumor cells and tumor-associated macrophages. T lymphocytes activation pathways are essential to immunotherapy including: target drug treatment, virus transfection, and adoptive cell transfer therapy.

#### 3.3.1 PD-1

Anti-PD-1 treatment has been a popular approach in clinical drugs, as it rescues T lymphocytes, particularly CD8^+^ T lymphocytes, from an immunosuppressive state and is specific in its action. Additionally, this treatment not only improves the anti-tumor ability but also increases the number of T lymphocytes, primarily through peripheral aggregation rather than proliferation within the brain microenvironment. The anti-PD-1 treatment has also been combined with other therapies to enhance the drug’s effect and elevate the number of CD44^+^CD62L-effector cells. It has been proposed that after stereotactic radiosurgery (SRS) or whole brain radiation therapy, immunotherapy including anti-PD-1 treatment can accelerate radiation-induced brain tissue changes by enhancing the immune microenvironment, as reflected by increased infiltration of T lymphocytes, mainly CD8^+^ T cells and reactive astrocytosis. However, it is been also reported that whole brain radiotherapy can sensitize breast cancer BrM to anti-PD-1 treatment in mouse models (Qian et al., 2011).

#### 3.3.2 Oncolytic viruses’

Oncolytic viruses (OVs) have recently emerged as a promising option in the anti-tumor field. OVs specifically target tumor cells as they are artificially modified. Upon infection of tumor cells, cytotoxic T cells are recruited to the infected lesion and kill the infected metastatic cells (Benencia et al., 2005; Gao et al., 2015; Du et al., 2017). Furthermore, OVs stimulate tumor cells to secrete IFN, which plays a key role in the PD-1/PD-L1 axis and promotes the expression of PD-L1 on metastatic cells. Research has shown that the effectiveness of anti-PD-1 therapy is determined by the expression of PD-L1 on the tumor, highlighting the importance of the PD-1/PD-L1 axis in treatment response (Steele et al., 2011).

Anti-PD-L1 treatment can have a more effective therapeutic action on metastases after OVs administration (Zhao et al., 2021). However, OVs immunotherapy is associated with virus-related toxicity (Benbenishty et al., 2019). Wanlu Du et al. invented oncolytic herpes simplex virus (oHSV)-armed mesenchymal stem cells (MSCs), which utilize MSCs as a virus carrier (Alomari et al., 2016; Taggart et al., 2018). This method avoids virus-related toxicity while keeping BBB penetration and high specificity. Clinically, OVs therapy has recently proven effective through intracarotid injection and local injection (Taggart et al., 2018).

#### 3.3.3 Adoptive cell transfer therapy

Adoptive cell transfer therapy (ACT therapy) includes various techniques such as chimeric antigen receptor-engineered T cell therapy (CAR T cell therapy), TIL therapy, and T cell receptor (TCR) therapy. These therapies have been applied clinically, but until recently, have not been widely tested in the context of BrM (Priceman et al., 2018). However, recent studies have shown that HER2+ breast cancer, NSCLC, and melanoma BrM are responsive to ACT therapy. For example, a study by Priceman et al. compared CD137 CAR T cells and CD28 CAR T cells in treating HER2+ breast cancer and found that CD137 CAR T cells had improved tumor killing effects, with reduced T-cell exhaustion and greater proliferative capacity (Priceman et al., 2018). Additionally, intraventricular injection was found to be as effective as intratumoral injection, offering a new administration option. Another study by Wang et al. used recombinant adeno-associated virus-denditic cells (DCs) to stimulate T lymphocytes and successfully limited tumor outgrowth in 3 patients (Wang et al., 2020). These DCs contained several specific tumor-associated antigenic determinant genes, including carcinoembryonic antigen (CEA), cytokeratin 19 (CK19), and prostate specific membrane antigen (PSMA), highlighting the potential of ACT therapies in treating BrM (Priceman et al., 2018; Wang et al., 2020). Recent study showed that CD19-targeted CAR T cells treat BrM not only through directly causing tumor cell apoptosis. Through single cell sequence, Parker, et al. found that brain mural cells which sustain integrity of BBB express CD19. When CD19-targeted CAR T cells attack mural cells and break BBB, cytokines can easily leak through the BBB and limit the BrM progression (Parker et al., 2020).

### 3.4 Targeting endothelial cells

Endothelial cells are a component of the BBB and play a crucial role in the extravasation of metastatic cells as well as giving metastatic cells several protections to promote tumor growth. The interaction between endothelial cells and metastatic cells can be divided into three parts, based on which drugs are designed (Haymaker et al., 2017).

First, metastatic cells can disrupt the inter-endothelial junction. MicroRNA (miR-101-3p), inhibits the cyclooxygenase-2 (COX-2)/MMP-1 pathway, where MMP-1 degrades the gap junction between endothelial cells. Loss of miR-101-3p therefore significantly increases the metastatic ability of metastatic cells. Experiments *in vitro* reported that restoring these microRNA in tumor cells decreased the metastatic burden in the brain (Niesel et al., 2021).

Secondly, metastatic cells cross the BBB through adhesion and metamorphosis. Adhesion is a vital step during this progress. In terms of tumor cells, α3β1 integrins can be elevated by αB-crystallin expressed in metastatic cells (Xiong et al., 2008). Silencing αB-crystallin through lentiviral and retroviral transduction *in vitro* blocked BrM progression (Malin et al., 2014). VCAM-1 is constantly expressed in endothelial cells, but significantly increased during inflammation (Marabelle et al., 2013). Hence, silencing these proteins on these 2 cells is proved to be effective in inhibiting BrM. microparticles of iron oxide (MPIOs)_-VCAM-1_, a VCAM-1 antagonist, succeed in limiting BrM progression in mice model (Sikpa et al., 2020).

Thirdly, after tumor cells extravasate into the brain stroma, tumor cells tend to stay close to endothelial cells for protection from immune cells and drugs. The gap junction between these 2 cells is essential for tumor growth. After the gap junction is established, endothelial cells receive IL-6 and IL-8 from tumor cells and secrete endothelin to tumor cells both through the paracrine pathway, resulting in an increased expression of survival protein in tumor cells (Benencia et al., 2005; Du et al., 2017). In this way, HER-2+ breast cancer BrM gains drug resistance against the targeted drug trastuzumab emtansine (T-DM1). Therefore, macitentan, a dual endothelin receptor (ETAR and ETBR) antagonist, is also applied, in order to inhibit this protective effect and can also be combined with a cytotoxic drug to increase drug effect (Gao et al., 2015). Furthermore, endothelial cells can secrete exosomes to tumor cells, resulting in an elevated expression of s100 calcium binding protein a16 (S100A16), which promotes prohibitin-1 (PHB-1) expression and inhibits apoptosis. siRNA inhibiting prohibitin-1 (PHB-1) to promote apoptosis of tumor cells is proved valid (Steele et al., 2011).

Additionally, some medicines are designed to transport more easily through the endothelial cells, thus acting more accurately on cancer cells. For instance, GRN1005, a novel conjugate of angiopep-2, a peptide facilitating brain penetration, and paclitaxel, is proved to be effective in endothelial transcytosis due to amplification of angiopep-2 on the surface of endothelial cells with BrM(Xu et al., 2019).

### 3.5 Targeting other cellular components of microenvironments

There are a variety of other cellular components that have been identified as potential targets in the microenvironment of BrM, but few studies have investigated these. For example, choroid plexus epithelial cells can be affected by complement component 3 (C3) produced by leptomeningeal metastases (LM) cells. C3 molecules act on C3a receptors on choroid plexus epithelial cells, resulting in the degradation of the intercellular tight junction. This permeabilization of the blood-brain barrier alters, allowing growth factors such as amphiregulin and other mitogens to enter and promote the growth of LM cells. C3aR inhibitor (SB290157) has been found to limit the growth of LM in mouse models (Boire et al., 2017). Additionally, neutrophils, which are PD-L1 positive, have been identified as immunosuppressive cells in BrM. Studies have shown that metastatic cells recruit immunosuppressive neutrophils through the Src/enhancer of zeste homolog 2 (EZH2)/c-JUN/granulocyte colony-stimulating factor (G-CSF) pathway, inhibiting the cytotoxic effect of CD8^+^ T lymphocytes. Inhibition of metastatic growth in mouse models has been achieved through blockade of G-CSF or Src (Zhang et al., 2020). Furthermore, research has also suggested that pericytes in the blood-brain barrier may have an anti-tumor role, but the underlying mechanisms are yet to be fully explored (Samson et al., 2018). What’s more, Jin, et al. found that microglia were the dominant cell population in NSCLC BrM microenvironment through single cell sequencing. They identified IL-6 as the key regulator in BrM cells to induce anti-inflammatory microglia *via* JAK2/STAT3 signaling, which in turn promoted the colonization process in BrM cells. IL6 inhibitors (tocilizumab or fedratinib) can block JAK2/STAT3 activation and impede BrM of NSCLC cells in mice model (Jin et al., 2022).

### 3.6 Summary

**FIGURE 1:**
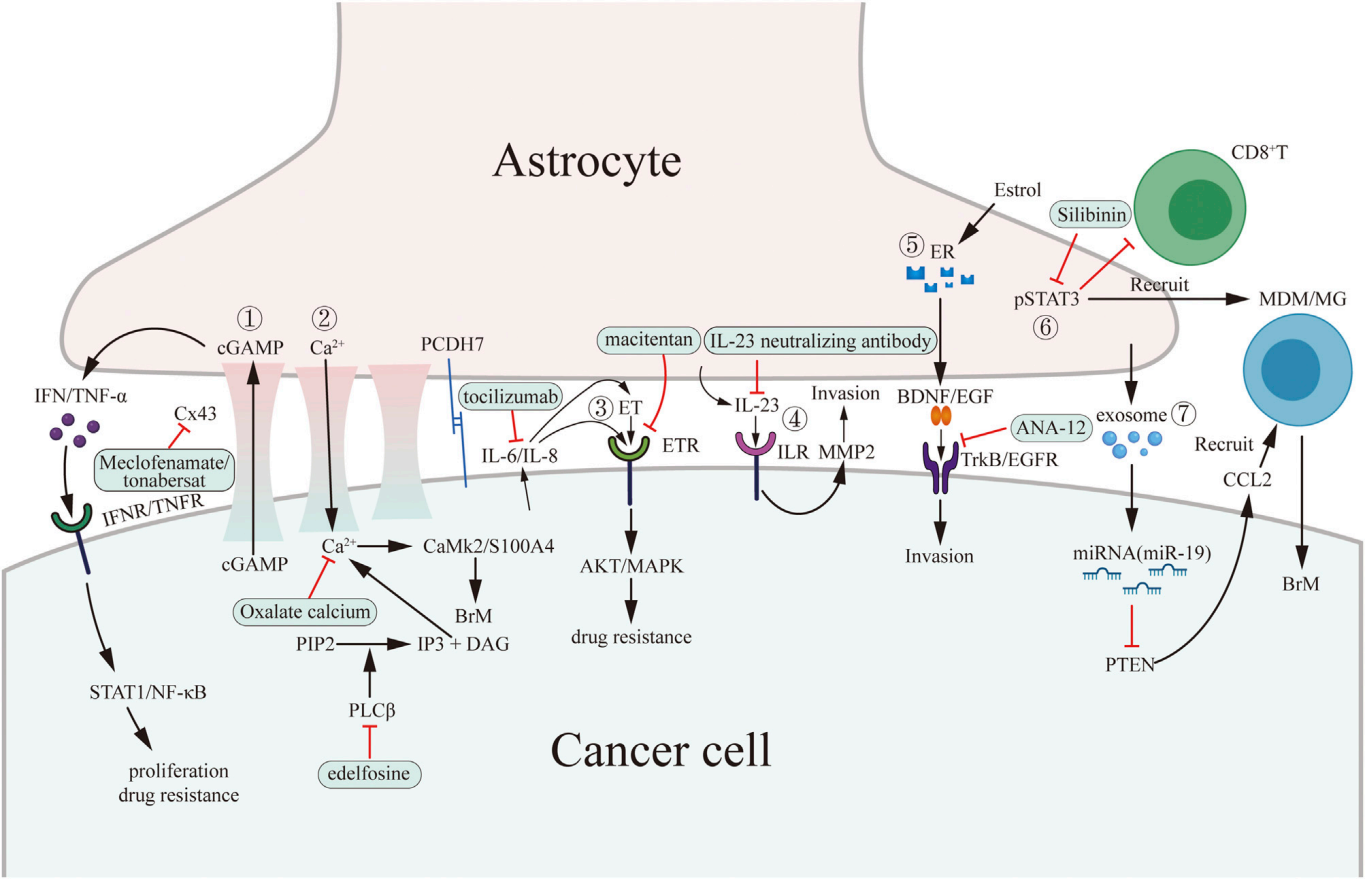
Pro-tumoral interaction between cancer cells and astrocytes and potential drug targets in brain microenvironment. Cancer cells interact with astrocytes through establishing gap junction, secreting signaling molecules (paracrine), secreting exosomes and regulating innate immunity and specific immunity by activating pSTAT3. The green boxes contain drugs used in experiments targeting microenvironment *in vitro* or *in vivo*. ①–③ belong to gap junction-dependent pro-tumoral interaction. ④–⑥ belong to paracrine-dependent pro-tumoral interaction. ⑦ belong to exosome-dependent pro-tumoral interaction.

**FIGURE 2:**
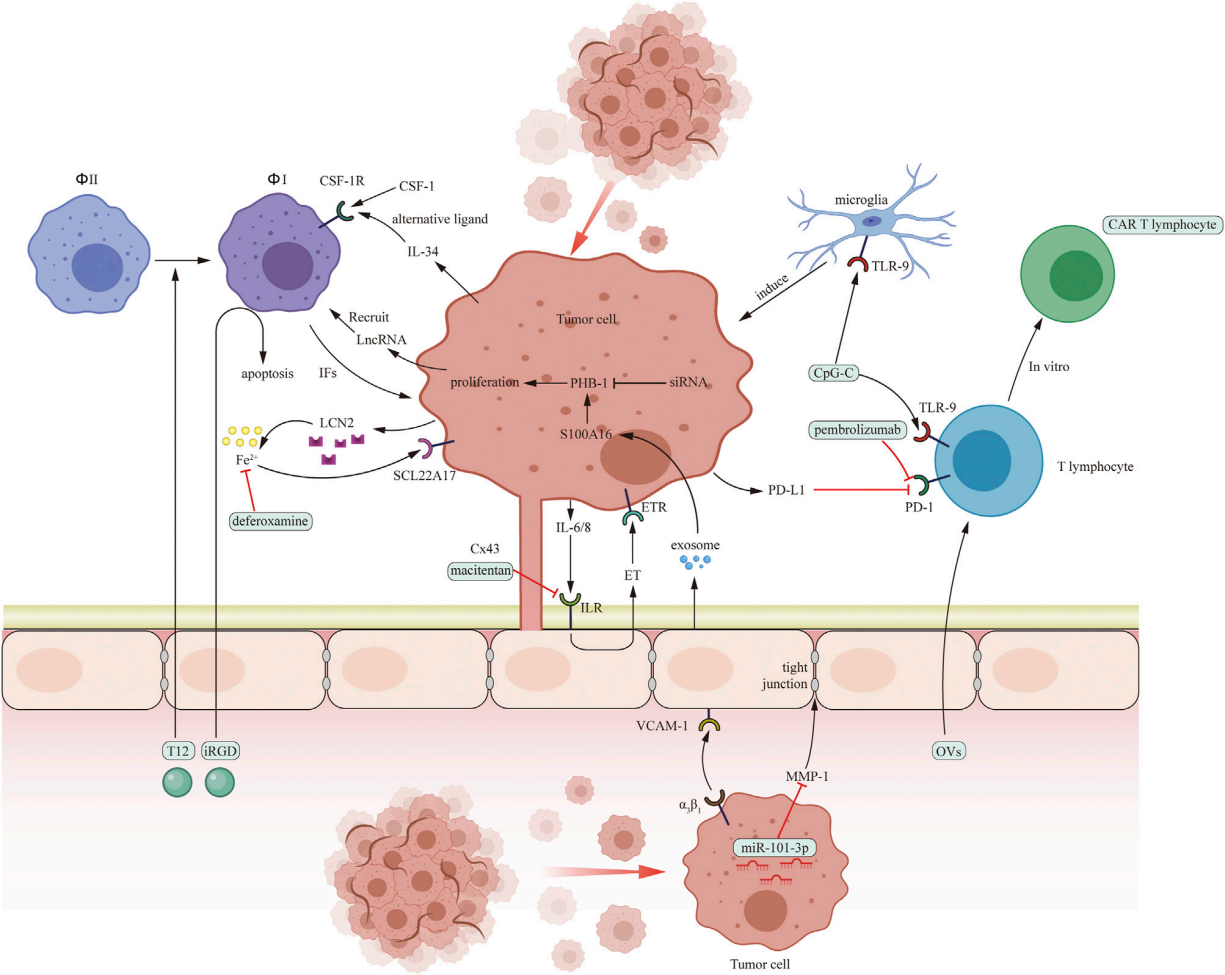
Pro-tumoral interaction between cancer cells and several cells in brain microenvironment including macrophages (*Φ*), microglia, T cells and endothelial cells and potential drug targets in brain microenvironment. The green boxes contain drugs used in experiments targeting microenvironment *in vitro* or *in vivo*.

In conclusion, current microenvironment-targeted therapies mainly focus on cellular component, including astrocytes, microglia, macrophages, T lymphocytes, and endothelial cells. Besides, we provide two figures to conclude all the mentioned pro-tumoral mechanisms and the corresponding targeted therapies (Figures 1, 2).

Tumor cells interact with astrocytes through gap junction, paracrine, exosomes, and STAT3 expression. Meclofenamate, tonabersat, edelfosine, and macitentan have the potential to disrupt the gap junction. Tocilizumab and ANA-12 inihibit IL-6 and TrkB paracrine pathway, respectively. Silibinin is an anti-STAT3 medicine. Besides, medicine targeting exosomes may be a potential therapeutic strategy.

Macrophages and microglia play an important role in BrM progression. PI3K, CSF-1, LncRNA, TLR9, and ionic iron are target molecule. Buparlisib and deferoxamine are PI3K and ionic iron inhibitors. In addition, nanocarriers targeting macrophages are proved to be effective *in vitro* and in mice model.

Several therapies targeting T lymphocytes include anti-PD-1 treatment (pembrolizumab and nivolumab), OVs therapy, and ACT therapy (CAR T cell therapy, TIL therapy, and TCR therapy).

Endothelial cells promote extravasation of metastatic cells in three steps. MPIOs-VCAM-1, macitentan, S100A16, and GRN1005 can inhibit one of these steps.

Also, SB290157 act on choroid plexus epithelial cells to inhibit metastatic progress. Therapies targeting other components are to be explored. Although some of the medicines are proved to be effective only *in vitro* or in mouse models, some are applied in clinical trials. Here, we provide a table concluding concurrent clinical trials which use microenvironment-targeted medicines (Table 5).

**TABLE 5:** Recent clinical trials on BrM microenvironment targeted therapies have been listed above. While medicines with new design conception are included, those commonly-seen medicines like VEGF inhibitor apatinib, CTLA-4 inhibitor ipilimumab, PD-1 inhibitor nivolumab, etc. Are not listed. Fulvestrant and tamoxifen citrate are estrogen receptor inhibitor. WP1066 is JAK2/STAT3 inhibitor. BKM120 is PI3K inhibitor. Endostar is recombinant VEGF inhibitor, QBS10072S LAT1-targeted medicine, GRN1005 a novel conjugate of angiopep-2 and paclitaxel, all of which are aim to transport through the BBB. The dendritic cell vaccine has been applied to other fields of cancer in the past, while until recently has it been tested in BrM. Microenvironment targeted therapies used here are marked red.

ANG1005 in breast cancer patients with Recurrent Brain metastases	Drug: GRN 1005	Completed	NCT02048059	Phase 2
ANG1005 in Leptomeningeal Disease From Breast Cancer	Drug: GRN 1005 Drug: Physician’s Best Choice	Not yet recruiting	NCT03613181	Phase 3
Expanded Access to ANG1005 for Individual Patients	Drug: GRN 1005	No longer available	NCT02755987	
Dose Escalation Study to Assess the Safety, Tolerability, Pharmacokinetics, and Pharmacodynamics of QBS10072S	Drug: QBS10072S	Recruiting	NCT04430842	Phase 1
Dendritic Cell Vaccines Against Her2/Her3, Cytokine Modulation Regimen, and Pembrolizumab for the Treatment of Brain Metastasis From Triple Negative Breast Cancer or HER2+ Breast Cancer	Biological: Anti-HER2/HER3 Dendritic Cell Vaccine Drug: Celecoxib Biological: Pembrolizumab Biological: Recombinant Interferon Alfa-2b Drug: Rintatolimod	Not yet recruiting	NCT04348747	Phase 2
A Phase II, Open-Label, Multicenter Study of Capmatinib in Subjects With MET Exon 14 Skipping Mutation Positive, Advanced, NSCLC With Brain Metastases	Drug: Capmatinib	Withdrawn	NCT04460729	Phase 2

## 4 Perspective

With a survival expectancy of patients is 17.6 months with single BrM and 17.9 months with multiple BrMs (Ferguson et al., 2012), BrM threaten the survival of cancer patients. Nowadays, current therapies cannot meet patients’ need to cure or prevent BrM. Microenvironment therapies provide a novel target with microenvironment cellular components leading to a complemented efficacy when combined with systemic treatments or other targeted therapies. Due to the lack of cytotoxic effects associated with microenvironment therapies, they typically do not have severe side effects unlike systemic chemotherapies or some targeted therapies. Furthermore, some microenvironment therapies enhance the efficacy of conventional drugs by reducing drug resistance and/or alternating the permeability of the BBB. Due to their indirect interaction pathway to inhibit tumor growth, whether these drugs can have a good response when clinically used is still not confirmed. Progress has been made in exploring mechanisms of interaction between metastatic cells and the brain microenvironment, drugs mentioned in these articles aim mainly at tumor cells instead of the microenvironment. Besides, the contribution of single cell sequence technology in the analysis, diagnosis, and treatment of BrM is promising and more researches about this approach are needed. Brain microenvironment deserves more concern due to its potential to cure or limit the outgrowth of BrM. Medicines targeting the microenvironment directly should be considered as novel therapeutics.

## Glossary

BrM: brain metastases;
BBB: blood-brain barrier;
BTB: blood-tumor barrier;
OS: overall survival;
HER-2: human epidermal growth factor receptor 2;
BRAF: B-Raf kinase gene;
MAPK: mitogen-activated protein kinase;
CTLA-4: cytotoxic T-lymphocyte-associated protein 4;
PD-1: programmed cell death protein 1;
PD-L1: programmed death-ligand 1;
ECM: extracellular matrix;
TNFRSF: tumor necrosis factor receptor superfamily;
PDCDILG2: programmed cell death 1 ligand 2;
IDO: indoleamine 2,3-dioxygenase;
ICOS: inducible T Cell costimulator;
CD: cluster of differentiation;
TARP: T cell receptor gamma alternate reading frame protein;
PTPRC: protein tyrosine phosphatase receptor type c;
PTPN7: protein tyrosine phosphatase non-receptor type 7;
IL10RA: interleukin 10 receptor subunit alpha;
GZMK: granzyme k;
CCR: C-C motif chemokine receptor;
CCL: C-C motif chemokine ligand;
BCL2L1: B-cell lymphoma-2-like protein 1;
TWIST1: twist family BHLH transcription factor 1;
GSTA5: glutathione S-transferase alpha 5;
TAMs: tumor-associated macrophages;
IL6R: interleukin 6 receptor;
INHBA: inhibin subunit beta a;
AREG: amphiregulin;
IDH: isocitrate dehydrogenase;
CNS: central nervous system;
NSCLC: non-small cell lung cancer;
TCR: T cell receptor;
STAT: signal transducer and activator of transcription;
Tim-3: T cell immunoglobulin and mucin-domain containing-3;
OXPHOS: oxidative phosphorylation;
KEGG: Kyoto Encyclopedia of Genes and Genomes;
E2F3: E2F transcription factor 3;
RB: retinoblastoma protein;
KRAS: Kirsten rat sarcoma virus;
ALK: anaplastic lymphoma kinase;
SRC: src proto-oncogene;
mTOR: mechanistic target of rapamycin kinase;
CXCR4: C-X-C motif chemokine receptor 4;
PLLP: plasmolipin;
TNFSF4: tumor necrosis factor superfamily member 4;
VCAM-1: vascular cell adhesion molecule 1;
SLC8A2: solute carrier family 8 member a2;
SLC7A11: solute carrier family 8 member a11;
TIL: tumor infiltrating lymphocytes;
S100A4: s100 calcium binding protein a4;
ANXA2: annexin a2;
TGFB1: transforming growth factor beta 1;
SERPINB1: serpin family B member 1;
ITGs: integrins;
PCDH7: protocadherin 7;
Cx43: connexin 43;
cGAMP: cyclic guanosine monophosphate–adenosine monophosphate;
IFN: interferon;
TNF-α: tumor necrosis factor alpha;
NF-κB: nuclear factor kappa b;
PLCβ: phosphodiesterase beta;
PIP2: phosphatidylinositol 4,5-bisphosphate;
IP3: inositol 1,4,5-trisphosphate;
DAG: 1,2-diacylglycerol;
CaMK2: calmodulin-dependent protein kinase II;
ETAR: endothelin receptor a;
ETBR: endothelin receptor b;
MIF: migration inhibitory factor;
IL-8: interleukin-8;
PAI-1: plasminogen activator inhibitor-1;
MMP: matrix metallopeptidase;
TNBC: triple-negative breast cancer;
BDNF: brain derived neurotrophic factor;
EGF: epidermal growth factor;
TrkB: tropomyosin receptor kinase b;
PTEN: phosphatase and tensin homolog;
SH2: src homology 2;
IBA1: ionized calcium binding adaptor molecule 1;
PI3K: phosphoinositide 3-kinase;
MDM: marrow-derived-macrophages;
CSF-1: colony stimulator factor-1;
**lncRNA**s: long non-coding RNAs;
JAK2: Janus kinase 2;
CpG-ODN: CpG oligodeoxynucleotides;
TLR: toll-like receptor;
iRGD-DMTPLN: iRGD-terpolymer-lipid hybrid nanoparticle with coloaded doxorubicin and mitomycin c;
LM: leptomeningeal metastases;
LCN2: lipocalin-2;
SCL22A17: solute carrier family 22 member 17;
SRS: stereotactic radiosurgery;
OVs: oncolytic viruses;
oHSV: oncolytic herpes simplex virus;
MSCs: mesenchymal stem cells;
ACT therapy: adoptive cell transfer therapy;
CAR T cell therapy: chimeric antigen receptor-engineered T cell therapy;
DCs: denditic cells;
CEA: carcinoembryonic antigen;
CK19: cytokeratin 19;
PSMA: prostate specific membrane antigen;
COX-2: cyclooxygenase-2;
MPIO: microparticles of iron oxide;
T-DM1: trastuzumab emtansine;
S100A16: s100 calcium binding protein a16;
PHB-1: prohibitin-1;
C3: complement component 3;
EZH2: enhancer of zeste homolog 2;
G-CSF: granulocyte colony-stimulating factor
